# PD64 Cost-Minimization Analysis And Psoriatic Arthritis: Insights From Health Technology Assessment Agency Recommendations

**DOI:** 10.1017/S0266462325101967

**Published:** 2025-12-29

**Authors:** Hérica Núbia Cardoso Cirilo, Aldenora Maria Ximenes Rodrigues, Ana Carolina Peçanha Antonio, Bruna Santos, Cecília de Oliveira Carvalho Faria, Alicia Dornelles, Ida Schwartz

## Abstract

**Introduction:**

Health technology assessment (HTA) bodies published stringent criteria for cost-minimization analyses (CMA), but evidence shows that many appraisals still fail to meet their full requirements: notably, adequately powered trials showing non-inferior outcomes regarding minimal clinically important difference. Here, we report the use of CMA in recommending biological disease-modifying antirheumatic drugs (bDMARDs) in psoriatic arthritis.

**Methods:**

A comprehensive review of HTA agency reports was conducted to identify the economic evaluations applied in the appraisal of bDMARDs for psoriatic arthritis. Data were collected from publicly available HTA databases and official websites of globally recognized agencies. The search included appraisals published up to December 2024. Evaluations were categorized based on the economic model used (e.g., CMA, cost effectiveness, cost utility, or cost benefit). Extracted data were analyzed to summarize trends and identify differences in the economic approaches used among agencies.

**Results:**

Cost-effectiveness and cost-utility analyses were the predominant approaches in HTA agency assessments of bDMARDs for psoriatic arthritis. Certolizumab pegol, golimumab, and secukinumab were the most frequently evaluated medications, each with nine evaluations. The Brazilian National National Committee for Health Technology Incorporation (CONITEC) was the only agency that exclusively used CMA in all its evaluations. In contrast, the National Institute for Health and Care Excellence (NICE) did not use this methodology in its assessments.

**Conclusions:**

Our findings highlight the significant variation in economic evaluation methods used by HTA agencies for bDMARDs in psoriatic arthritis. The inappropriateness of superiority trials that fail to reject the null hypothesis for CMA is a key takeaway. While CMA remain relevant due to their simplicity, their use by CONITEC contrasts with broader methodologies preferred by other agencies, particularly NICE.

